# A Case of Cleidocranial Dysplasia with a Novel Mutation and Growth Velocity Gain with Growth Hormone Treatment

**DOI:** 10.4274/jcrpe.galenos.2018.2018.0211

**Published:** 2019-09-03

**Authors:** Emine Çamtosun, Ayşehan Akıncı, Emine Demiral, İbrahim Tekedereli, Ahmet Sığırcı

**Affiliations:** 1 İnönü University Faculty of Medicine, Department of Pediatric Endocrinology, Malatya, Turkey; 2 İnönü University Faculty of Medicine, Department of Medical Biology and Genetics, Malatya, Turkey; 3 İnönü University Faculty of Medicine, Department of Radiology, Malatya, Turkey

**Keywords:** Cleidocranial dysplasia, RUNX2, severe short stature

## Abstract

Cleidocranial dysplasia (CCD) is a rare congenital autosomal dominant skeletal disorder that is characterized by hypoplasia or aplasia of clavicles, failure of cranial suture closure, dental anomalies, short stature and other changes in skeletal patterning and growth. The gene responsible for pathogenesis has been mapped to the short arm of chromosome 6p21, core binding factor alpha-1 (*CBFA1*) or runt related transcription factor-2 (*RUNX2*). Here we describe a CCD patient with a novel mutation in the *RUNX2* gene. A five-and-a-half year old girl presented with severe short stature, dysmorphic facial appearance (hypertelorism, prominent forehead, high palate, midfacial hypoplasia), macrocephaly, large anterior fontanelle, increased anteroposterior chest diameter. Her shoulders were close to each other and her bilateral clavicles appeared short on physical examination. Bilateral hypoplastic clavicles, coxa valga, hypoplasia of iliac bones, wide symphysis pubis and phalangeal dysplastic features were detected on her skeletal X-ray examination. She was diagnosed as having CCD. Molecular analysis detected a novel heterozygous mutation ‘NM_001024630.3p.T155P(c.463A>C)’ in the *RUNX2* gene. At age seven years and two months old, because of her severe short stature, growth hormone (GH) treatment was started and she responded well to GH therapy with no adverse effects. In conclusion, hypoplasia or aplasia of the clavicles, failure of cranial suture closure, dental anomalies and short stature should bring CCD to mind. We present a novel mutation in the *RUNX2* gene for CCD. We obtained growth velocity gain with GH treatment in our patient.

**What is already known on this topic?**

Classical cleidocranial dysplasia (CCD) is characterised by hypoplasia or aplasia of clavicles, failure of cranial suture closure and dental anomalies. Short stature is a frequent feature of the syndrome. Nearly two hundred mutations associated with CCD have been reported.

**What this study adds?**

We present a likely novel mutation for CCD. Although data about growth hormone (GH) therapy for CCD with severe short stature is very limited, we observed a gain in growth velocity with GH treatment.

## Introduction

Cleidocranial dysplasia (CCD) (CCD; OMIM:119600) is a skeletal dysplasia characterized by hypoplasia or aplasia of the clavicles permitting abnormal facility in apposing the shoulders, by persistently open skull sutures with bulging calvaria and by dental anomalies including delayed exfoliation of primary teeth, delayed eruption of permanent teeth and multiple impacted supernumerary teeth. Short stature, generalized bone dysplasia, vertebral malformations, a depressed nasal bridge and a wide pubic symphysis can also be seen (1). The estimated prevalence of CCD is one per million births, which is most likely underdiagnosis, and there is no sex predilection (2).

CCD is caused by heterozygous loss-of-function mutation in the runt related transcription factor-2 *(RUNX2)* gene, encoding the transcription factor core binding factor alpha-1 *(CBFA1)* on chromosome 6p21 (1,3). The human *RUNX2 (CBFA1)* gene consists of eight exons and it controls differentiation of precursor cells into osteoblasts and is essential for membraneous and endochondral bone formation (3,4). It is a master regulatory gene for skeletal development and morphogenesis. The majority of *RUNX2* mutations in classic CCD patients are missense or nonsense mutations. Frame shift and exon skipping mutations (4), insertions and deletions have also been described (3). The disease is commonly autosomal dominantly inherited but can be sporadic.

Here we present a CCD patient with significant short stature with typical characteristics of CCD and a novel *RUNX2* mutation. She had growth hormone (GH) therapy with growth velocity gain.

## Case Report

**Figure 1:**
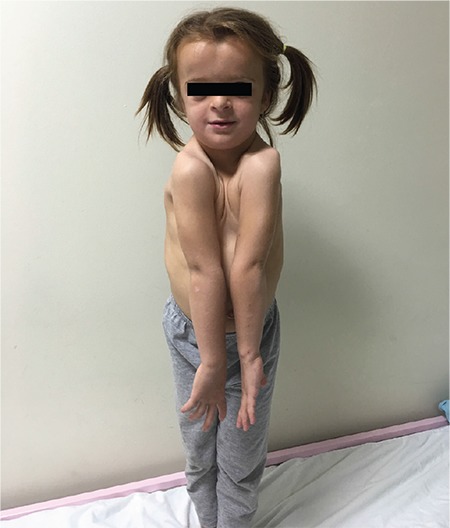
Hypoplasia of the clavicles permitting abnormal facility in apposing the shoulders

A five-and-a-half year old girl was admitted to our hospital due to her short stature and dysmorphic features. Her anthropometric measurements and standard deviation (SD) scores (SDS), according to Turkish standards (5), were as follows: height was 94.3 cm (-3.69 SD); weight was 13.7 kg (-2.45 SD); body mass index (BMI) was 15.4 (-0.05 SD); head circumference was 52 cm (0.77 SD); upper/lower segment ratio of 1.25 (>+2 SD); and mid parental target height was 161.15 cm (-0.31 SD). The parents had no history of constitutional delay of puberty and growth. The patient had a dysmorphic face with hypertelorism, a prominent forehead, high palate, midfacial hypoplasia and down-slanting palpebral fissures. In addition she had macrocephaly, large anterior fontanelle, increased anteroposterior chest diameter, laxity in her distal joints and pes planus. Her shoulders were close to one another and her clavicles appeared too short (Figure 1). Exfoliation of her primary teeth was delayed. She had normal developmental milestones and intelligence, except for a mild speech delay. Her neurological examination was normal.

**Figure 2:**
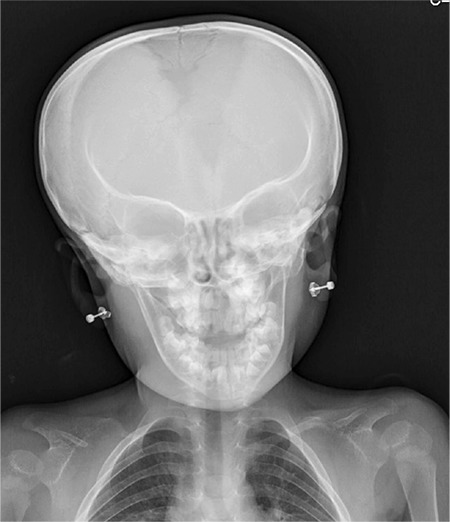
Skull X-ray of patient shows wide and open anterior fontanelle

**Figure 3:**
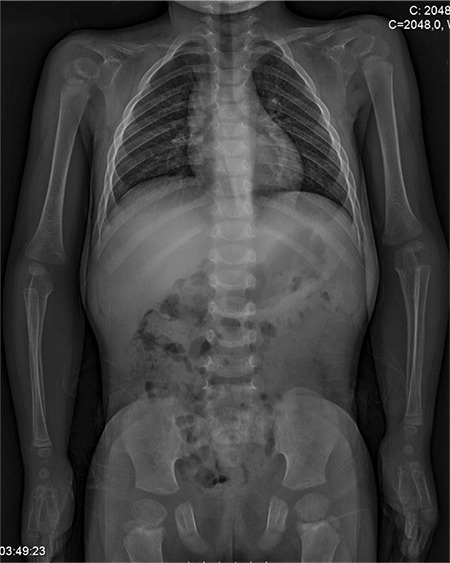
X-ray of her trunk shows bilateral hypoplastic clavicles, hypoplasia of iliac bones, wide symphysis pubis

Bone age was 3-3.5 years according to the method of Greulich and Pyle. Skeletal X-rays showed bilateral hypoplastic clavicles, a wide and open anterior fontanelle, coxa valga, hypoplasia of iliac bones and a wide symphysis pubis (see Figures 2, 3). Her hand X-ray examination revealed cone shaped epiphyses, a pseudo-epiphysis of the second metacarpal, tapering of distal phalanges, severe dysplasia of the middle phalanx in the fifth finger and a wide phalangeal epiphysis. These findings were compatible with the diagnosis of CCD. She had no scoliosis.

In laboratory studies her blood count, biochemical tests, thyroid function tests and urine examination results were normal. Tissue transglutaminase antibodies were negative. Insulin like growth factor-1 (IGF1) and IGF binding protein-3 (IGFBP3) concentrations were 74 ng/mL (-1.15 SD) and 2860 ng/mL (-0.12 SD) respectively. Her peak GH concentration following L-DOPA stimulation was 13.4 ng/mL (non-deficient). Karyotype was 46,XX.

**Figure 4:**
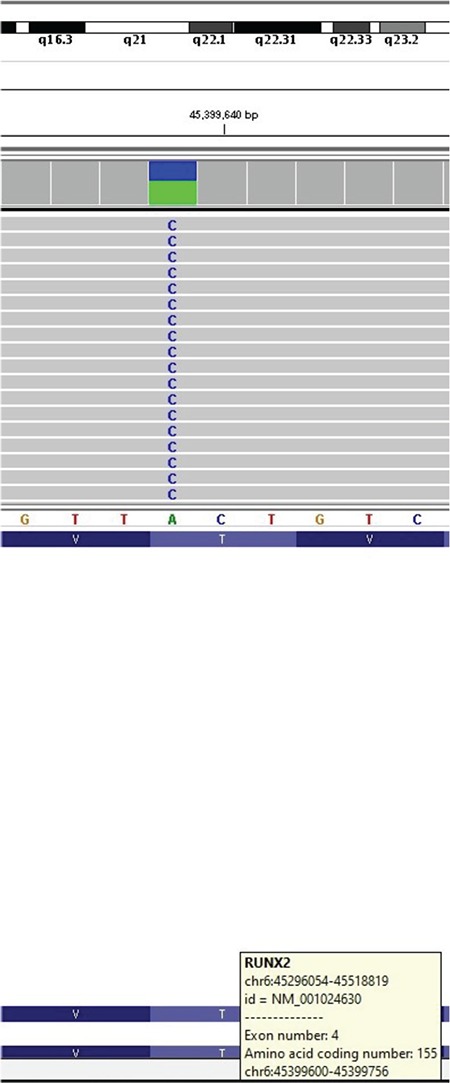
MISEQ sequence image of the mutation in *RUNX2* gene

After genetic consultation, next generation sequencing (NGS) detected a novel heterozygous mutation ‘NM_001024630.3p.T155P(c.463A>C)’ in the *RUNX2* gene (Figure 4). *RUNX2* gene sequence analysis was performed by using MiSeq NGS platform, an FDA approved diagnostic system (Illumina Inc., San Diego, CA, USA). Genomic DNA was extracted according to the manufacturer’s standard procedure using the QIAamp DNA Blood Midi Kit (Qiagen, Hilden, Germany). All coding exons of the *RUNX2* gene and their flanking splice site junctions were amplified using polymerase chain reaction (PCR) primers, designed with PRIMER©-Primer Designer v.2.0 (Scientific and Educational Software programme) software. PCRs were validated by using agarose gel electrophoresis. After PCR amplification, the libraries were prepared with the Nextera XT kit (Illumina Inc., San Diego, CA, USA), according to the manufacturer’s instructions. Next-gene sequencing was carried on MiSeq (Illumina Inc., San Diego, CA, USA). Sequences were aligned to the hg19 genome within MiSeq Reporter software (Illumina Inc., San Diego, CA, USA). Visualisation of the data was performed with IGV 2.3 (Broad Institute, Cambridge, MA, USA) software.

This mutation has not been reported previously and it is highly likely to be pathogenic according to the PolyPhen-2 (score=1.00, sensitivity: 0.00, specificity: 1.00) (http://genetics.bwh.harvard.edu/pph2), SIFT (score=0.0001 converted rank score=0.912), Provean (score=-5.46 -5.53 converted rankscore=0.86) and Mutation Taster (score=0.99) software analysis. Her mother’s genotype was normal for this mutation. It was not possible to perform the father’s genetic analysis.

At age seven years and two months old, the patient’s anthropometric characteristics were: height 104.1 cm; height SDS -3.8 SD; body weight 17.1 kg (-2.3 SD); and upper/lower segment ratio 1.28. Her bone age was estimated as five years.

An IGF generation test was performed with 0.1 mg/kg/day GH for four days because of her severe short stature. The test revealed a 200% increase in IGF1. Subcutaneous GH treatment was started at a dose of 30 mcg/kg/day. After one year of treatment (at age 8 years and three months) her growth velocity was found to have increased to 8.2 cm/year from 5.28 cm/year before treatment. Height SDS had increased to -3.15 SD. She was still prepubertal and her bone age was 6.5-7 years. Her IGF1 concentration was 123 ng/mL (-0.03 SD) and IGFBP3 concentration was 5460 ng/mL (1.08 SD) after GH treatment. She was followed-up every three months and no adverse side effects were observed which could be associated with GH treatment. She was also followed by an orthopedist for pes planus and a pediatric dentist for delayed exfoliation of primary teeth. She continues to receive GH therapy. After 21 months of GH therapy, at age 9 years, she was prepubertal and her anthropometric measures were as follows: height 119.2 cm (-2.28 SD); weight 22.6 kg (-1.48); BMI 15.9 (-0.26 SD); upper/lower segment ratio 1.16 (>+2 SD) (+2 SD=1.08); and arm span 115 cm. Her body disproportion had not worsened.

A written informed consent was obtained from the patient’s family regarding the scientific publication of the patient’s photographs and her medical data.

## Discussion

CCD is generally diagnosed clinically and the diagnosis is supported by radiography. Genetic analysis reveals a *RUNX2* mutation in almost 70% of patients. Our patient was diagnosed due to her classical phenotypic and radiological features.

Short stature can be a feature of CCD due to generalized bone dysplasia. Reports of gender differences and severity of short stature are controversial (2,6,7). Short stature is usually mildly disproportional but it also can be proportional (2,7). Studies which include younger CCD patients indicate that birth lengths are normal, but heights decrease to around -2 SD at ages of 4-8 years (6).

Jensen (6) investigated somatic development in 17 Danish CCD patients, aged 5-46 years. Stature was documented in six males and eight females and compared with Danish reference data. The report noted growth retardation, especially in females. Heights of CCD males were clustered between the 5^th^ and 50^th^ percentiles, but heights in all CCD females were below the 5^th^ percentile (between -1.81 SD and -3.56 SD). Due to the small number of patients, having no data about parental heights and noting that four females belonged to the same family, the authors concluded that the observed severity of short stature in females may have occurred by chance. It was also noted that the females had smaller head circumference values than the boys (-0.77 SD versus +0.27 SD, respectively).

Our female patient had a significant short stature and her height SDS was significantly lower than her mid-parental height SDS. Her head circumference was relatively macrocephalic (head circumference +0.77 SD).

In the study of Cooper et al (2), 21 female and 21 male CCD patients aged >18 years were evaluated for height. The authors observed that their patients had shorter statures than their healthy relatives. The mean height percentiles of girls and boys were 10^th^ percentile (38% were <5^th^ percentile) and <5^th^ percentile (62% were <5^th^ percentile), respectively. Unlike Jensen’s (6) report, short stature in this study was more prominent in males and severe short stature was not observed among the cases. Most prevalent skeletal deformities of the cases were genu valga and pes planus.

Dinçsoy Bir et al (7) reported 15 CCD cases in 11 independent families. Short stature was observed in three males (height SDS were -4.2, -2.9 and -2.24) and a female (height SDS was -2.55) and all were proportional. Three of these short patients had low IGF1 levels (<-2 SD). The female patient showed partial GH deficiency on GH stimulation tests and had normal hypophyseal magnetic resonance imaging. She had not received GH therapy at the time of the report. The male patient with a height SDS of -4.2 did not have low IGF1 and no GH deficiency was found. He did not respond to one year of GH therapy at the age of 15 years, but his bone age was not reported in the study.

Short stature of different frequency and severity, reported in CCD patients, can be explained with the variety in the effects of the known mutations. There are studies investigating genotype-phenotype correlations for *RUNX2* mutations (4,8). Yoshida et al (8), studied genotype-phenotype correlations in 17 Japanese CCD patients and they reported that *RUNX2* mutations which affect the Runt domain (responsible for binding to DNA) are correlated with short stature and its severity. They showed that patients had normal stature when they had mutations with an intact Runt domain. The mutation detected in our patient [‘NM_001024630.3p.T155P(c.463A>C)’] was a missense mutation leading to a change in 155^th^ amino acid in Exon 4 and located within the Runt domain. It was likely to be pathogenic in the *in silico* analysis. This situation can explain our patient’s severe short stature.

Yoshida et al (8), found that short stature and the number of supernumerary teeth were correlated significantly. Different studies have shown mutations which affect the Runt domain of the *RUNX2* gene also cause the classical CCD phenotype and are associated with severe dental anomalies (4). Genotype-phenotype correlation studies also showed that mutations of the *RUNX2* gene could lead to various phenotypic features even in the same family (4).

Data on GH treatment for CCD patients are very limited. In the study of Dinçsoy Bir et al (7), one patient with CCD who was treated with GH for one year did not benefit from this treatment but the patient was 15 years old and his bone age data were missing. Our patient’s height increased by 8.2 cm/year (prepubertal), an increase of 3 cm/year more than in the year prior to GH treatment. This corresponded to an increase in height SDS of 0.65 SD/year. The initiation of GH therapy at an early age could have been the reason for the better outcome observed in this patient. However, in terms of efficacy and safety of GH therapy, there is a need for randomized controlled trials involving more patients.

It has been suggested that there can be increased bone fragility in CCD patients. Cooper et al (2) reported two patients with multiple bone fractures, but they found similar fracture and osteoporosis rates in 90 CCD patients and in the control group. Dinçsoy Bir et al (7) reported that more than 50% of their patients had osteoporosis and they also reported no relationship between osteoporosis in their patients and vitamin D deficiency. Our patient had no bone fractures and the radiology of her vertebrae showed no fractures or scoliosis.

In conclusion, we report a patient who presented with severe short stature, failure of cranial suture closure and hypoplasia of clavicles, who was diagnosed as having CCD. A novel mutation in the *RUNX2* gene for CCD was detected. We obtained a growth velocity gain with GH treatment for severe short stature, with no side effects. Randomized controlled trials are necessary however, for evaluating the effectiveness and safety of GH therapy for this population.
